# Pollen Season Trends (1973-2013) in Stockholm Area, Sweden

**DOI:** 10.1371/journal.pone.0166887

**Published:** 2016-11-29

**Authors:** Tomas Lind, Agneta Ekebom, Kerstin Alm Kübler, Pia Östensson, Tom Bellander, Mare Lõhmus

**Affiliations:** 1 Institute of Environmental Medicine, Karolinska Institutet, Sweden; 2 Centre for Occupational and Environmental Medicine, Stockholm County Council, Sweden; 3 Department of Environmental Research and Monitoring, Swedish Museum of Natural History, Sweden; Oklahoma State University, UNITED STATES

## Abstract

In the present study, the phenological and quantitative changes in the pollen seasons between 1973 and 2013 in the Stockholm region of Sweden were studied for nine types of pollen (hazel, alder, elm, birch, oak, grass, mugwort, willow and pine). Linear regression models were used to estimate the long term trends in duration, start- and end-dates, peak-values and the yearly accumulated pollen sums of the pollen seasons. The pollen seasons of several arboreal plant species (e.g. birch, oak and pine) were found to start significantly earlier today compared to 41 years earlier, and have an earlier peak-date, while the season of other species seemed largely unaffected. However, the long term trends in the end-dates of pollen seasons differed between arboreal and herbaceous species. For herbaceous species (grass and mugwort), a significant change towards later end-dates was observed and the duration of season was found to have increased. A significant trend towards an earlier end-date was found in the majority of the arboreal plant species (i.e. elm, oak, pine and birch), but the length of the season seemed unaffected. A trend towards an increase in yearly concentrations of pollen was observed for several species; however the reasons for this phenomenon cannot be explained unambiguously by the present study design. The trend of increasing yearly mean air temperatures in the Stockholm area may be the reason to changed phenological patterns of pollen seasons.

## Introduction

Global average temperatures have increased since the 1950’s and are predicted to rise further in the coming decades [1]. This increase in temperature is affecting the phenology of flowering plants, including the timing of pollen release into the air. Accordingly, in several parts of central and northern Europe, advances in the seasonal patterns of plant phenology, including the timing for pollen release, have been observed [2,3]. These phenological changes have a wide range of consequences for ecological processes, agriculture, forestry, and also for human health [4].

A number of European studies report changing seasonal trends in birch (*Betula* spp.) pollen during the last three or four decades [5–9]. In Copenhagen, for example, the birch pollen season started about 14 days earlier, the peak-date arrived 17 days earlier and the season ended 9 days earlier in year 2000 compared to the year 1977 [9]. In Basel, Switzerland, it was shown that due to a temperature increase, the start of birch flowering occurred about 15 days earlier in 2006 than in 1969 [6]. Analyses from UK indicate that birch pollen seasons have advanced with approximately 5 days per decade since 1970’s [8]. However, recent analyses by Newnham et al. [10] did not show any change in timing in birch pollen season in UK during 1995–2010. Several studies report also a trend to increased concentrations of birch pollen [5,6,11–13]. In addition to birch, several European studies have shown changes over time in pollen season characteristics of i.e. grass (Poaceae) [14], oak (*Quercus*) [15], alder (*Alnus*) [16], and several weed taxa (*Artemisia* spp., *Rumex* spp. and Poaceae and Urticaceae species) [17].

Birch is considered to be the top-ranked producer of aero-allergenic pollen in NW Europe, however, several other types of pollen contribute to allergic responses [18] and other health outcomes, such as the lung function in children [19]. According to the data from Palynological Laboratory at Swedish Museum of Natural History (http://pollenrapporten.se), in central Sweden, deciduous trees, such as hazel (*Corylus*) and alder are the earliest seasonal producers of allergenic pollen. Depending on meteorological conditions, these species may begin to release pollen as early as late February and early March in Stockholm area. Birch typically flowers between mid-April and early June followed by the oak in May-June. The native pollen season in Scandinavia usually ends when grass (the major cause of pollinosis in central and southern Europe) and mugwort (*Artemisia vulgaris*) have stopped flowering in September. The typical producers of grass pollen in Sweden are the tall meadow grasses such as timothy (*Phleum pratense*), orchard grass (*Dactylis glomerata*) and meadow foxtail (*Alopecurus pratensis*).

In the Stockholm region, Sweden, continuous monitoring of airborne pollen was started in 1973 by the Palynological Laboratory, Swedish Museum of Natural History. Since then, the results of daily pollen monitoring for species such as alder (*Alnus*), hazel (*Corylus*), elm (*Ulmus*), willow (*Salix*), birch (*Betula*), oak (*Quercus*), grass (Poaceae) and mugwort (*Artemisia*) have been reported to the public. These pollen types are present in central Sweden and considered to be of clinical importance. In this study, the changes in the timing of the pollen seasons between 1973 and 2013 for nine types of pollen were studied with special focus on the start, end, peak-date and duration of the season. The scope of the paper does not include an investigation of associations between specific underlying climatic events and the pollen characteristics, but solely focuses on the trends over time. In addition to eight pollen types previously mentioned, pine (*Pinus*) was included since it is one of the most frequent pollen types.

## Material and Methods

The location of the study was the city of Stockholm with a population approximately 1.6 million. This capital has a humid continental climate with about 1800 hours of sunshine per year. The biome of area belongs to the temperate deciduous forest. Stockholm is surrounded by 219 nature reserves, and approximately 30% of the city’s area is covered with vegetation. Alder, birch, oak, hazel, aspen (*Populus tremula*), willow, pine and spruce (*Picea abies*) are the domineering groups of native trees. However the city has about 12 000 trees planted in street environments and other groups and species, such as linden (*Tilia* spp.), maple (*Acer* spp.), horse chestnut (*Aesculus hippocastanum*), cottonwood (*Populus* spp.) and Swedish whitebeam (*Sorbus intermedia*), are common as ornamental trees (http://www.stockholm.se/KulturFritid/Park-och-natur/Trad/). According to the data from Swedish Meteorology and Hydrology Institute (SMHI), the average yearly temperature of Stockholm is +6.6°C (1973–2015, http://www.smhi.se). February is the coldest month of the year with an average temperature of -2.6°C, and July the warmest with an average temperature of +17.1°C.

Pollen sampling was performed with Burkard volumetric pollen and spore trap based on the Hirst design [20]. Airborne particles were deposited on sticky tape mounted on a drum, which was slowly turned by clockwork. The tape was embedded in stained glycerine gelatine and analyzed under an optical light microscope using x 400 magnification. The pollen of different taxa were determined and their numbers were counted according to the 12 transversal transects method [21]. The sampling method produced the pollen count expressed as concentration in pollen grain/m^3^/24 hours.

By using this method, pollen data has been collected in Stockholm since 1973, thus covering a period of 41 years. From 1973 to 1993 the trap was located in a central part of Stockholm, 20 meters above ground level (59°20'49.9"N 18°2'56.9"E). From 1993 to 2013 the pollen trap was located on the roof of a building about 15 meters above ground level at Stockholm University Campus, north of Stockholm city (59°21'56.2"N 18°3'34.7"E). The distance between the two locations is about 2 km. During one year, the monitoring was performed parallel at the two locations and no major differences were observed (data not published). Thus, the two sampling places were considered to be very similar. The purpose of placing the trap on the roof of a building is to avoid the distortion of the pollen count by local pollen emissions.

Daily pollen counts/m^3^ of hazel (Co), alder (Al), elm (Ul), birch (Be), oak (Qu), grass (Po), mugwort (Ar), willow (Sx) and pine (Pi) from 1973–2013 were analyzed in the present study. The total yearly accumulated pollen count was expressed as the sum of all daily counts per year. The start and end dates of the season were defined as the dates upon which the accumulated sum of daily pollen counts/m^3^ reached 3% and 97% respectively of the total pollen produced. This interval (3%-97%) was found to capture the local pollen season most accurately and best eliminated the pollen originating from long-distance transport. The duration of the season was defined as the number of days with accumulated counts between 3% and 97%. The peak-date was defined as the date with the highest daily count. When several dates had the same daily concentration values, the earliest of the dates was used as the peak-date. Years where the yearly total production of pollen by a genus was < 50 counts/m^3^/season were excluded from the analysis of start-, end- and peak-dates and duration (In total, three years were excluded from trend analyses of Co, and one year of Qu and Ul).

Linear regression models (separate models for each pollen type) were used to estimate the long-term trends in duration, start- and end-dates, peak-values and the yearly accumulated pollen sums, of the pollen seasons between 1973 and 2013. The explanatory variable ‘year’ was centered towards the start year 1973 (i.e., 1973 = year 0). Regression models for yearly total sum of pollen were based on log-transformed data and the result was checked for influential years using criteria Cooks D > 4/N and dfbeta > 2/$\sqrt{N}$ [22]. Influential years were identified according to these criteria for all pollen species and, for each pollen type the most influential year was excluded. To illustrate the background air temperature trends during the corresponding time period, temperature data measured at Bromma airport located 8 km from Stockholm city center were retrieved from the SMHI web page http://www.smhi.se/klimatdata/meteorologi/temperatur/1.2847. The long term temperature trend was estimated using linear regression based on yearly averages. All calculations were made using Stata v 13 software (StataCorp LP, TX, USA).

## Results

**Fig 1:**
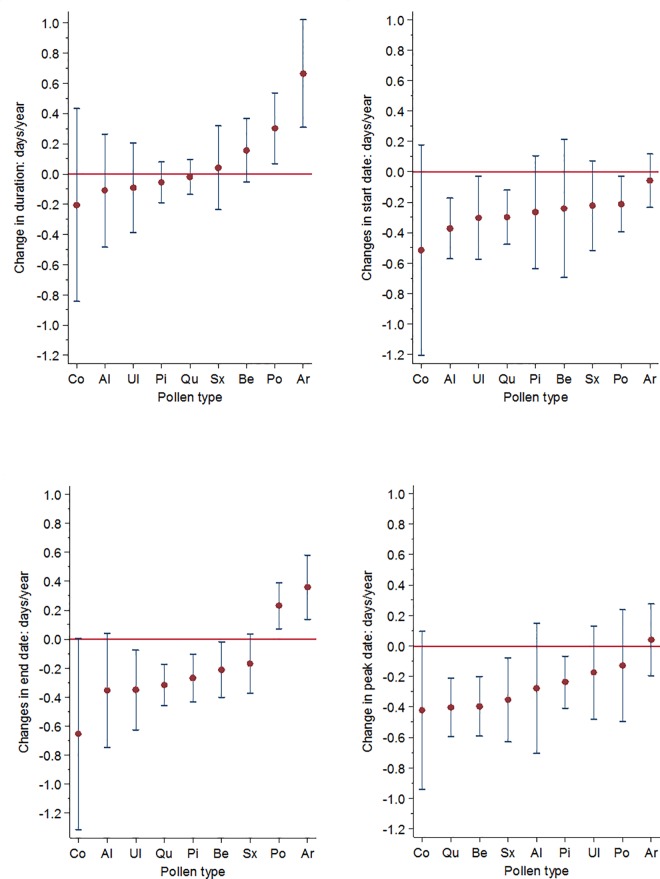
The change in pollen season characteristics per year for hazel (Co), alder (Al), elm (Ul), Pine (Pi), oak (Qu), willow (Sx), birch (Be), grass (Po), and mugwort (Ar) a) Duration b) Start-date c) End-date d)Peak-date. Error bars depict 95% confidence interval.

**Fig 2:**
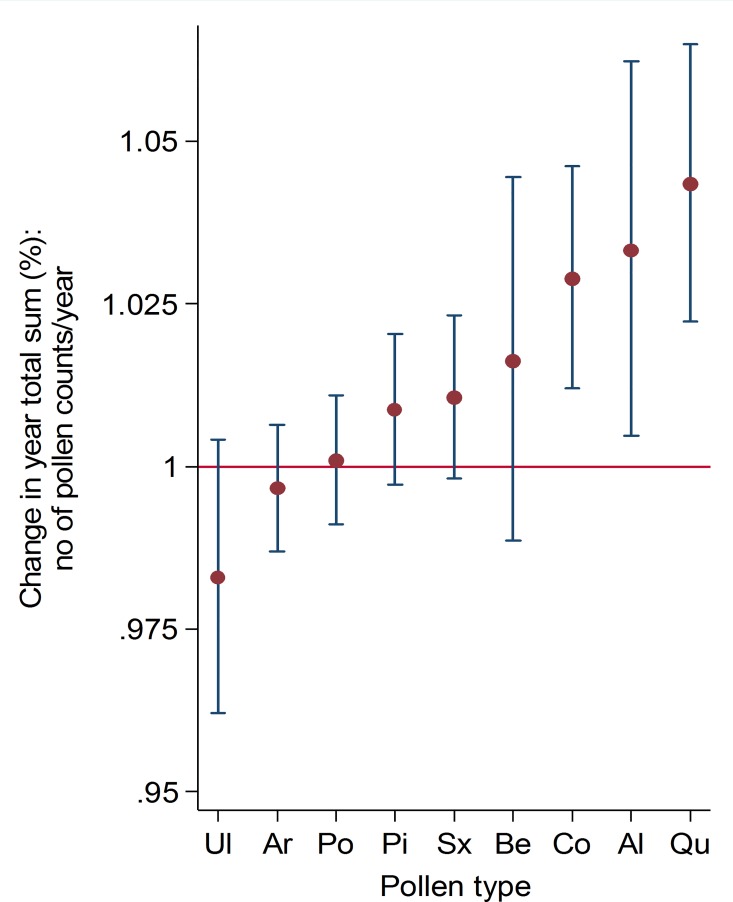
The proportional change/year in total yearly pollen counts. Error bars depict 95% confidence interval.

**Fig 3:**
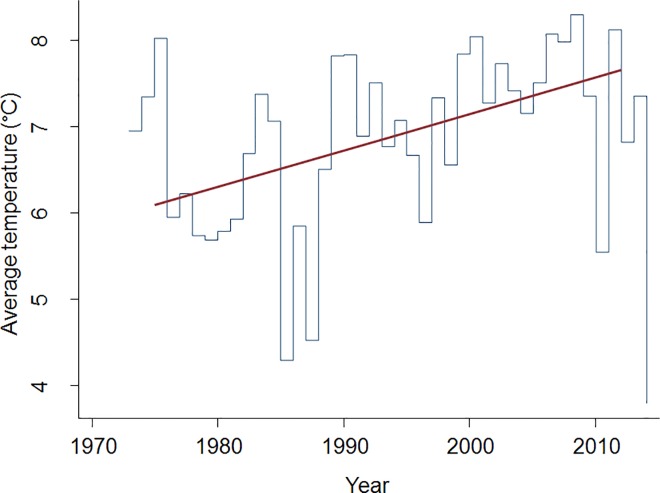
Average yearly temperatures in the Bromma measuring station, Stockholm area 1973–2013 (Swedish Meteorology and Hydrology Institute).

Changes in the duration of the pollen seasons for all pollen types over the period 1973 to 2013 are shown in Fig 1A. In herbaceous plants that are blooming relatively late in the season, such as grass and mugwort, the duration of the pollen seasons showed a statistically significant increase at 95% confidence interval with +0.30 days/year (i.e. +12 days/41 years) and +0.66 days/year (i.e. +24 days/41 years) respectively (Table 1). No other pollen types showed significant changes in the season length. Changes in the start dates for all pollen types for years 1973–2013 are shown in Fig 1B. Based on a linear model, birch, pine, oak and mugwort showed statistically significantly earlier start dates in the beginning of the study period compared to the end of the study period (a change of -0.37; -0.21, -0.30, and -0.30 days/year respectively at 95% confidence interval; Table 1). Changes in the end dates (Fig 1C) differed between arboreal and herbaceous plant species. While elm, oak, pine, and birch showed significant earlier end dates of the pollen season (a change of -3.5, -0.32, -0.27, and -0.21 days/year respectively at 95% confidence interval), the herbaceous plants, mugwort (+0.36 days/year) and grass (+0.23 days/year) had significantly later end dates of the pollen season at the end of the study period compared to the beginning (Table 1). The peak dates (Fig 1D) arrived significantly earlier in the end of the study period compared to the beginning in birch, oak, willow and pine (a change of -0.40, -40, -0.35, and -0.24 days/year respectively, at 95% confidence interval; Table 1). The change in percentage of the total yearly accumulated pollen count showed an increasing trend in several species. The percentage of increase, however, was only significant for hazel, alder and oak (Fig 2). The linear fit to average values of the yearly air temperature for Bromma, Stockholm for the same time period, is shown in Fig 3.

**Table 1:** Pollen trends 1973–2013. The significant trends (CI>95%) are marked with **bold** style. The negative sign in front of a value indicates a trend towards an earlier start-date, peak-date or end-date, a shortening trend in the duration of the pollen season or quantities of pollen produced. Positive numbers indicate a trend towards a later start-date, peak-date or end-date, a lengthening trend in the duration of the pollen season and increased quantities of pollen produced. DOY indicates the no of day of year. The 25 and 75 percentile values for all pollen types were determined by first calculating the average number of pollen grains/m^3^/day for each season and thereafter obtaining the average percentile values across the whole 41 year period.

Genus		Season characteristics				Pollen quantities		No of days with pollen concentrations…		
		Duration (days)	Start (DOY)	End (DOY)	Peak (DOY)	Year total (grains/m^3^)	Max/day (grains/m^3^)	below 25 percentile	between 25 and 75 percentile	over 75 percentile
**Alder (*Alnus*)**	**Mean**	30.83	79	109	91.58	1150.59	241.24	27.22	9.34	3.76
	**SD**	13.93	17.08	15.13	16.16	1430.20	333.51	9.96	6.22	4.04
	**Min**	13	38	63	55	68	11	12	0	0
	**Max**	76	104	153	117	8110	2032	57	31	15
	**R2**	0.01	0.03	0.08	0.04	0.00	0.00	0.07	0.02	0.04
	**Slope**	-0.11	-0.24	-0.35	-0.28	3.05	-1.12	0.22	0.07	0.07
	**p**	0.560	0.290	0.080	0.197	0.870	0.803	0.100	0.380	0.185
	**41-year trend**	-4.51	-9.84	-14.35	-11.38	125.05	-45.86	9.02	2.87	2.91
**Mugwort (*Artemisia*)**	**Mean**	41.34	197.43	238.00	216.28	190.07	22.61	15.73	13.07	7.59
	**SD**	15.41	10.73	9.27	8.66	69.57	12.14	6.55	4.12	4.17
	**Min**	25	161	226	180	87	8	4	5	1
	**Max**	83	211	266	241	389	57	28	23	18
	**R2**	**0.27**	**0.12**	**0.21**	0.00	0.00	0.00	**0.41**	0.00	0.02
	**Slope**	**0.66**	**-0.30**	**0.36**	0.04	0.06	0.05	**0.35**	0.02	-0.05
	**p**	**0.001**	**0.030**	**0.002**	0.737	0.950	0.780	**0.000**	0.678	0.338
	**41-year trend**	**27.06**	**-12.30**	**14.76**	1.60	2.46	1.87	**14.35**	0.90	-2.19
**Birch (*Betula*)**	**Mean**	28.30	119.22	146.61	127.37	8471.20	1473.63	62.66	9.05	4.51
	**SD**	7.97	8.62	7.52	8.67	7386.31	1473.79	13.84	5.44	4.52
	**Min**	16	92	129	110	314	53	39	0	0
	**Max**	51	138	161	149	27099	6109	93	25	13
	**R2**	0.06	**0.27**	**0.11**	**0.28**	0.03	0.01	**0.14**	0.01	0.02
	**Slope**	0.16	**-0.37**	**-0.21**	**-0.40**	101.61	14.55	**0.43**	0.05	0.05
	**p**	0.140	**0.001**	**0.030**	**0.000**	0.300	0.461	**0.017**	0.527	0.401
	**41-year trend**	6.56	**-15.17**	**-8.61**	**-16.40**	4166.01	596.62	**17.54**	1.89	2.09
**Hazel (*Corulus*)**	**Mean**	34.34	75.93	108.68	87	126.61	24.12	8.80	6.54	4.61
	**SD**	20.01	21.68	21.33	19.35	62.01	19.61	4.43	3.82	3.12
	**Min**	9	38	74	46	56	1	0	0	0
	**Max**	106	105	186	122	309	94	21	17	15
	**R2**	0.02	0.08	0.14	0.07	**0.22**	0.06	0.05	0.04	**0.13**
	**Slope**	-0.21	-0.51	-0.65	-0.42	**2.37**	0.41	0.08	0.06	**0.09**
	**p**	0.510	0.140	0.052	0.107	**0.013**	0.110	0.173	0.213	**0.021**
	**41-year trend**	-8.61	-20.91	-26.65	-17.30	**97.17**	16.99	3.28	2.65	**3.84**
**Pine (*Pinus*)**	**Mean**	25.34	146.49	170.90	152.85	8693.63	1459.59	73.29	5.10	5.24
	**SD**	5.04	7.26	6.86	6.93	3662.55	794.82	13.52	2.91	3.26
	**Min**	15	125	157	131	2179	317	39	1	0
	**Max**	41	159	185	165	15416	3653	104	10	11
	**R2**	0.02	**0.12**	**0.22**	**0.17**	0.04	0.01	**0.18**	0.01	**0.09**
	**Slope**	-0.06	**-0.21**	**-0.27**	**-0.24**	62.45	5.87	**0.48**	-0.02	**0.08**
	**p**	0.410	**0.024**	**0.002**	**0.008**	0.200	0.582	**0.006**	0.539	**0.052**
	**41-year trend**	-2.30	**-8.73**	**-11.07**	**-9.84**	2560.45	240.56	**19.52**	-0.98	**3.40**
**Grass (*Poaceae*)**	**Mean**	73.61	157.73	230.90	181.32	1159.15	85.51	59.78	11.90	17.27
	**SD**	9.39	6.60	6.49	13.65	461.28	61.09	11.55	3.93	6.78
	**Min**	54	145	217	146	220	16	43	5	0
	**Max**	93	174	248	206	2523	353	89	23	32
	**R2**	**0.15**	0.01	**0.18**	0.01	0.01	0.03	**0.60**	0.01	0.02
	**Slope**	**0.30**	-0.06	**0.23**	-0.13	3.49	-0.83	**0.74**	0.04	0.07
	**p**	**0.013**	0.510	**0.005**	0.481	0.570	0.307	**0.000**	0.475	0.411
	**41-year trend**	**12.30**	-2.46	**9.43**	-5.29	143.07	-34.18	**30.50**	1.54	3.06
**Oak (*Quercus*)**	**Mean**	15.18	140.33	154.50	145.61	1754.85	424.78	22.07	4.68	2.54
	**SD**	4.15	7.29	6.30	8.64	1332.28	409.48	6.77	2.85	2.42
	**Min**	7	120	143	129	142	4	11	0	0
	**Max**	24	152	168	169	6386	2075	41	10	7
	**R2**	0.00	**0.23**	**0.35**	**0.31**	**0.35**	**0.29**	**0.17**	**0.19**	**0.32**
	**Slope**	-0.02	**-0.30**	**-0.32**	**-0.40**	**66.49**	**18.43**	**0.23**	**0.10**	**0.11**
	**p**	0.752	**0.002**	**0.000**	**0.000**	**0.000**	**0.000**	**0.008**	**0.005**	**0.000**
	**41-year trend**	-0.78	**-12.30**	**-13.12**	**-16.40**	**2726.25**	**755.44**	**9.48**	**4.21**	**4.62**
**Willow (*Salix*)**	**Mean**	40.27	106.51	145.93	117.34	374.61	51.05	22.32	11.27	7.22
	**SD**	10.28	11.23	7.76	11.05	158.95	28.43	7.08	4.04	4.23
	**Min**	25	78	130	90	71	10	10	5	0
	**Max**	65	130	159	146	650	123	38	21	16
	**R2**	0.00	0.06	0.07	**0.15**	**0.12**	0.02	**0.09**	0.04	**0.11**
	**Slope**	0.04	-0.22	-0.17	**-0.35**	**4.53**	0.37	**0.18**	0.07	**0.12**
	**p**	0.771	0.136	0.099	**0.013**	**0.029**	0.336	**0.054**	0.195	**0.036**
	**41-year trend**	1.64	-9.02	-6.93	**-14.35**	**185.73**	14.99	**7.34**	2.86	**4.77**
**Elm (*Ulmus*)**	**Mean**	19.33	107.00	125.38	112.83	899.58	221.44	22.44	4.27	3.05
	**SD**	10.91	14.02	10.93	11.45	675.06	214.79	7.34	3.85	2.70
	**Min**	9	59	92	77	63	13	8	0	0
	**Max**	57	131	148	134	2764	1019	46	20	10
	**R2**	0.01	0.05	**0.15**	0.03	0.08	0.07	0.03	0.01	0.02
	**Slope**	-0.09	-0.26	**-0.35**	-0.17	-15.38	-4.89	-0.12	-0.04	-0.04
	**p**	0.531	0.157	**0.014**	0.253	0.086	0.085	0.273	0.462	0.327
	**41-year trend**	-3.73	-10.66	**-14.34**	-7.16	-630.58	-200.34	-4.76	-1.56	-1.45

## Discussion

The phenology and characteristics of the pollen seasons in Stockholm area have been changing during the last four decades, possibly, due to the increasing temperatures in the area (Fig 3). Nevertheless, the trends of these changes are not uniform across all types of pollen. A general trend towards an earlier start of the pollen season was more or less uniformly observed in most types of pollen. However, the duration and the end-date of the seasons showed different trends depending on whether the pollen came from arboreal or herbaceous plant species. The duration of the pollen season significantly increased in the herbaceous species (grass and mugwort) whereas no significant changes in the season duration were detected in the arboreal ones. The end-dates of the season showed a trend towards an earlier end date in the majority of the arboreal plant species, whereas a later end date was observed in the herbaceous species. These changes in the pollen seasons are likely to influence the timing of allergy outbreaks in sensitized population in the Stockholm area.

Previous studies have shown a clear correlation between the pre-season air temperatures and the start- and peak-dates of tree pollen season [9,11–13]. In the present study, no correlations between pollen and temperature data were carried out. However, according to the data from the SMHI, the average air temperatures for the Stockholm area are about 1°C higher now than they were in 1970’s (Fig 3). Furthermore, the vegetation period in southern Sweden is presently more than 10 days longer than it was in 1970’s (http://www.smhi.se/klimatdata/meteorologi/temperatur). Consequently, it is plausible that the changes in climate characteristics are behind the earlier start- and peak-dates of pollen seasons today compared to 1970’s.

Changes in the temporal distribution of airborne pollen contribute directly to the patterns of allergic airway diseases and associated public health costs [23]. A prolonged pollen season increases the duration of human exposure to aeroallergens, and can therefore increase the risk for allergic sensitization. For persons already sensitized to pollen, a longer season is likely to increase the duration of allergy symptoms [24]. In addition, as annual concentrations and peak values of pollen are predicted to increase under climate change scenarios, and are in some cases already increasing [5,6,25], it may also lead to more severe allergic symptoms [24]. The prevalence of pollen allergy is increasing in many countries of Europe, [18,26]. In Switzerland for example the prevalence of hay fever increased from 4.8% in 1958 to 16.1% in 1999 [27]. A Danish study reported an increase in pollen allergy from 6.5% in 1987 to 10.3% in 1997 [28], whereas another study from the same country showed an increase in pollen allergy prevalence of about 50% between 1989 and 1997 [29]. In 2009, approximately 30% of the Stockholm population reported to be allergic to pollen, and the self-reported prevalence of hay fever increased from 15% in 1997 to 23% in 2007 in the same region [30]. Several hypotheses, including the hygiene hypothesis and the possible role of air pollution and land use change have been discussed as the underlying reasons [27,31,32], but so far there is no definite explanation to this development and the role of changing pollen seasonality is so far unexplored.

Longer pollen seasons of grass and mugwort compared to forty years ago were identified in this study and may lead to increase the suffering in sensitized people and hence add to the related medical costs. A relatively high number of different species belonging to the family of Poaceae contribute to the pollen type grouped under the name “grass”. Consequently, “grass” has a very long season, and may affect pollen sensitized people during a period from May to September. Grass allergens mostly induce nasal and conjunctival symptoms but may also exacerbate asthma by inducing an inflammatory response involving T-cells, mast cells and eosinophils [33]. In addition, the mugwort that blooms from late July to September produces a very potent type of pollen with high antigen activity [34]. Only 6–12 counts/m^3^ of mugwort pollen are needed (compared to, for example, a minimum of 30 counts/m^3^ of grass pollen and around 80–100 counts/m^3^ of birch pollen), for a sensitized person to develop symptoms of allergy [34].

The total annual pollen count is an important quantitative index that directly correlates to the increased prevalence of allergy symptoms and increased healthcare and financial costs [25]. An increase in the amount of pollen produced has been shown to result from exposure to increased surrounding temperature and CO_2_ concentrations–both of which are characteristic of the present observed climate change [18]. However, according to two Mediterranean studies, different biotypes of species, are expected to respond to changing environmental factors in different ways–while some arboreal plats are observed to show increasing trends in their annual pollen counts, the herbaceous species in Mediterranean regions are, instead, showing decreasing trends [35,36]. A trend towards an increase in the annual total amount of birch pollen and higher peak-values of concentrations has been reported in earlier European studies [5,6,12]. In the present dataset, however, the inter-year variation of birch pollen production was high and we did not detect any significant trends in the total annual birch pollen counts.

A significant proportional increase in the annual total of produced pollen was found in hazel, alder and oak. Nevertheless, since in the present study the vegetation and land-use changes in the Stockholm area were not analyzed, the quantitative pollen trends can have three plausible explanations: a) the genus has changed its quantitative pollen production; b) the number of plants belonging to a particular genus has changed over the last 41 years in the Stockholm area, or possibly c) more of the plants from the specific genus are now growing in close proximity to the pollen trap. By placing the pollen trap on a roof top, the local micro-environmental effects (explanation c) are believed to be minimized; still, a possible local influence cannot be excluded completely from the discussion.

The proportional increase in the hazel, alder and oak pollen in Stockholm area may have a clinical relevance. Because of allergenic cross-reactivity, the early-blooming trees, such as hazel and alder, can act as primers for the allergic sensitization to birch pollen allergens and increase clinical symptoms during birch-pollen season [18]. Oak, following the birch pollen season, can additionally prolong the season of tree pollen allergy [18]. A proportional increase in the total yearly pollen concentrations of hazel, elm and oak therefore has the potential to increase the severity of the clinical symptoms in sensitized people in the area. The decreasing (albeit non-significant) trend in the yearly elm pollen count could be explained by the elimination of elm trees as part of efforts to hinder the spread of the Dutch elm disease, *Ophiostoma ulmi*, which was discovered in Stockholm in 1998 (http://www.stockholm.se/KulturFritid/Park-och-natur/Trad/Almsjuka/).

Pollen monitoring usually starts prior to the expected start-date for hazel and alder. As the weather has a considerable impact on flowering and thus pollen release, the start-date for these two species can vary from year to year, especially for hazel. Because it is often impossible to let the pollen trap run for the whole winter, the initial days of pollen production and consequently the start-date of the pollen season can be missed. This is likely to affect the credibility of the data and add to the inter-year variation of the start- and peak-dates, as well as the yearly total pollen count values of very early species such as the hazel and alder. The end-dates of the pollen season have most likely been adequately estimated in most years.

Our study has shown, as have others that pollen seasons are starting earlier today than for four decades ago for a number of species. However, the long-term trends in the end dates of pollen seasons differed between arboreal and herbaceous species. Furthermore, since 1973, the duration of the pollen season has only been increasing in herbaceous species. Consequently, due to an earlier season start in the arboreal species and a later end-date of the herbaceous species, the total period of pollen exposure has been increasing since 1973. A trend towards an increase in yearly concentrations of pollen was observed for several species; however the reasons for this phenomenon cannot be explained unambiguously by the present study. A more detailed study, taking into account inter-annual, month-to-month and diurnal cycle variabilities in meteorological, phonological and biological factors may lead to better prediction of pollen production.
